# Effects of Vitamin D on the Renin–Angiotensin System and Acute Childhood Pneumonia

**DOI:** 10.3390/antibiotics11111545

**Published:** 2022-11-03

**Authors:** Andrea Zovi, Francesco Ferrara, Roberta Pasquinucci, Livia Nava, Antonio Vitiello, Roberto Arrigoni, Andrea Ballini, Stefania Cantore, Raffele Palmirotta, Marina Di Domenico, Luigi Santacroce, Mariarosaria Boccellino

**Affiliations:** 1 Ministry of Health, Viale Giorgio Ribotta 5, 00144 Rome, Italy; 2 Pharmaceutical Department, Asl Napoli 3 Sud, Dell’amicizia Street 22, Nola, 80035 Naples, Italy; 3 Pharmaceutical Department, Asl Napoli 2 Nord, Frattamaggiore, 80027 Naples, Italy; 4 CNR Institute of Biomembranes, Bioenergetics and Molecular Biotechnologies (IBIOM), 70124 Bari, Italy; 5 Department of Precision Medicine, University of Campania “Luigi Vanvitelli”, 80100 Naples, Italy; 6 Independent Researcher, Regional Dental Community Service “Sorriso&Benessere-Ricerca e Clinica”, 70129 Bari, Italy; 7 Interdisciplinary Department of Medicine, School of Medicine, University of Bari “Aldo Moro”, 70129 Bari, Italy

**Keywords:** vitamin D, renin–angiotensin system, infectious disease, antibiotics, pharmacology, pneumonia

## Abstract

Vitamin D promotes kidney calcium reabsorption and regulates calcium and phosphate metabolism, as well as the intestinal absorption of calcium and phosphorus and bone mineralization events. Vitamin D is also known for its immunomodulatory properties. It has been shown in the literature that the active form of vitamin D, 1,25-dihydroxyvitamin D, performs multiple functions in the adaptive and innate immune system, as well as acting on the endothelial membrane. Recent evidence shows that vitamin D is a negative endocrine modulator of the renin–angiotensin system (RAS), with protection from diseases leading to lung damage, such as pneumonia caused by various pathogens. Vitamin D support associated with the use of antibiotics could be crucial to counteract these infectious diseases.

## 1. Introduction

Vitamin D is a hormone with multiple functions, among which extraskeletal and skeletal functions are distinguished. There are several variants of vitamin D with biological and physiological properties, and they are vitamin D2 (ergocalciferol), derived mainly from plants and plant sources, and vitamin D3 (cholecalciferol), synthesized by the body. Cholecalciferol is synthesized in the epidermis (basal layers) from cholesterol by the action of sunlight and ultraviolet radiation. Cholecalciferol is incorporated into chylomicrons and is drained by the lymphatic system, thus entering the venous blood. Vitamin D can be taken up and originate from the skin or from the diet and is biologically inert, so initial hydroxylation in the liver by vitamin D-25-hydroxylase (25-OHase) to 25(OH)D is required. Subsequently, 25(OH)D requires further hydroxylation in the kidneys by 25(OH)D-1-OHase (CYP27B1) to arrive at the biologically active form of vitamin D 1,25(OH)2D [1]. 1,25(OH)2D stimulates, in enterocytes, the production of TRPV6, a calcium transporter, and calbindin, an intracellular calcium-binding protein [2]. Without vitamin D, only 60% of calcium and phosphorus are absorbed [3]. The vitamin D receptor belongs to the large cholecalciferol receptor family of thyroid and steroid hormone receptors. It is expressed by cells in many organs, such as the brain, heart, and skin; it is a transcription factor. The receptor with vitamin D forms a heterodimer with the retinoid X receptor (RXR) by forming a bond with the hormone response receptor. In this manner, it binds the hormone response element to DNA, modulating the expression of a specific gene. In humans, the receptor is encoded by the VDR gene, while the DNA sequence to which VDR–calcitriol–RXR binds is a hormone-response element. VDRE is a special DNA sequence placed in a gene promoter that binds with a complex, VDR–RXR–calcitriol. VDRE can also be called a hormone-response element, because VDR is a member of the hormone receptor family [4].

## 2. The Role of Vitamin D in the Skeletal and Immune Systems

The maintenance of plasma calcium levels and bone mineral homeostasis is the main effect of vitamin D. VDR, the vitamin D receptor, is present in most human tissues, and particularly in tissues at the level of osteocytes, osteoblasts, and osteoclasts [5]. A lack of VDR receptor function or lack of active metabolite Vit D causes rickets, an impaired mineralization of bone tissue, in children [6,7]. Active metabolite Vit D with its own VDR receptor is responsible for calcium absorption in the intestines and kidneys [8]. All VDR activities are critical for calcium absorption, which is also critical for the regulation of bone mineral homeostasis within bone cells. Studies in in vitro and in vivo models have produced evidence that vitamin D activity can stimulate bone formation or inhibit bone formation by acting on bone catabolism. These actions on bone minerals are supported by the maintenance of plasma calcium homeostasis under various physiological conditions [9]. In addition to its effects on the bone skeleton, active metabolite Vit D has several extraskeletal functions, such as immunomodulatory functions. In various diseases, such as respiratory infections, influenza, bacterial diseases, and even viral infections or fungal diseases, active metabolite Vit D deficiency is a potential factor related to the progression of the disease [10]. Vitamin D causes the differentiation of monocytes into macrophages, is responsible for the activation of the inducible nitric oxide synthetase (iNOS) oxidative pathway, an important anti-pathogenic mechanism, and the phosphatidyl inositol 3-kinase (PI3K) signaling pathways that generate reactive oxygen species (ROS). Active metabolite Vit D also modulates the expression of pattern recognition receptors (PRRs) in monocytes, including tool-like receptors TLR4 and TLR2. A reduction in the expression and subsequent decrease in pathogen-associated molecular pattern (PAMP) signaling is necessary to reduce the inflammatory process triggered by the human innate immune system [11]. Furthermore, vitamin D, when taken, reduces the proliferation of interleukin IL-12 and IL-23 and acts directly on antigen-presenting T and B lymphocytes in an antigen-presenting cell (APC) cell-independent manner. Active metabolite Vit D may alter the cytokine profile and subsequent proliferation of T lymphocytes through the inhibition of the cytokines IL-2, IL-17, and IL-21, TNF-α, and IFN-γ, increasing the level of the cytokines IL-4, IL-5, and IL-10 [12]. It is noteworthy that interference with the renin–angiotensin system (RAS) could mediate several of the main effects of vitamin D.

## 3. Acute Childhood Pneumonia

### 3.1. Pathology

Among children under the age of five, infantile pneumonia is the major cause of developing severe illness and premature death [13]. Clinically, this is an acute respiratory infection due to lower respiratory tract inflammation, mainly affecting the lungs. The lungs consist of small sacs called alveoli, which, in the presence of an inflammatory process, can fill with exudative fluid, which results in impaired breathing, gas exchange in the lungs, increased lung resistance, and limited oxygen supply [14]. Bacteria and viruses are the main causative factors of the development of childhood pneumonia; in some situations, there is a risk that the microbial infection may transform into more severe disease due to the action of additional opportunistic bacteria that have colonized the lungs, developing a superinfection. The illness can lead to the proliferation of innumerable bacterial organisms, such as Streptococcus pneumoniae, which is the most common in preschool and school-age children; Mycoplasma pneumoniae, Chlamydia pneumoniae, Haemophilus influenzae type-B; and Staphylococcus aureus, which is the main offender in severe lung infections [15,16]. Major risk factors include mixed feeding, non-breastfeeding resulting in poor nutrition, indoor air pollution, prematurity, and low birth weight. Overcrowding and lack of immunization against measles (within the first 12 months of life) may also contribute to a predisposition toward pneumonia [17,18]. Childhood pneumonia, depending on some factors, such as the child’s health status, age, the microbial agent involved, and severity of the disease, may itself manifest clinically differently. These include cough, fever, difficulty breathing with wheezing, tachycardia, and increased respiratory rate [19].

### 3.2. Antibiotics

According to World Health Organization (WHO) guidelines, pneumonia is classified into severe pneumonia, pneumonia, or non-pneumonia, and treatment is tailored based on this classification, which includes an assessment of the clinical symptoms and severity of the illness. Severe pneumonia requires hospitalization and parenteral antibiotics, pneumonia requires treatment with oral antibiotics and outpatient management with follow-up, while non-pneumonia in children with coughs and colds is treated symptomatically [13,14,15,16,17,18,19]. A Cochrane review published in 2018 assessed the efficacy and safety of vitamin D supplementation in association with antibiotics for the treatment of acute childhood pneumonia, providing new evidence that needs additional information [17]. The management of acute childhood pneumonia includes antibiotics, oxygen, supportive therapy, and assisted ventilation (in severe cases) [10]. The choice of empiric antibiotic treatment depends on the child’s age, possible etiology, antimicrobial resistance patterns, previous treatments, as well as factors affecting host susceptibility, including HIV, nutritional status, and vaccination status [20]. Oral amoxicillin is the first-line therapy in children with rapid-breathing pneumonia without signs of chest pain and general signs of danger: at least 40 mg/kg/dose twice daily (80 mg/kg/day) for five days. Amoxicillin provides adequate coverage against most pathogens that cause community-acquired pneumonia, including Streptococcus pneumoniae, the most important invasive bacterial pathogen. If first-line therapy fails, children with rapid-breathing pneumonia should be carefully managed in a dedicated facility to be treated with second-line therapy. Oral amoxicillin at a minimum dosage of 40 mg/kg/dose twice daily for five days is indicated for patients aged 2 to 59 months with thoracic pneumonia. Patients aged 2 to 59 months who present symptoms of severe pneumonia should receive, as a first-line treatment, the administration of ampicillin (or benzyl penicillin) and gentamicin parenterally in combination (ampicillin: 50 mg/kg or benzyl penicillin: 50,000 units per kilogram of IM/EV every 6 h for at least five days; and gentamicin: 7.5 mg/kg IM/EV once daily for at least five days). The second line of treatment in this case involves ceftriaxone. A macrolide, such as clarithromycin or azithromycin, may be associated with the prevention of M. pneumoniae or C. pneumoniae infection [21,22]. Symptomatic therapy includes the use of analgesic drugs, such as acetaminophen and ibuprofen, and the maintenance of adequate hydration [23].

## 4. Renin–Angiotensin System and Infectious Disease

The renin–angiotensin system (RAS) is a physiological system that can regulate several body actions, among which the most important are blood pressure regulation, circulating plasma volume (called volemia), and arterial muscle tone. The RAS is actually a cascade of enzymatic actions: the classic and main one involves renin converting angiotensinogen, released by liver cells, into angiotensin I (Ang I). Ang I is then converted to angiotensin II (Ang II) by angiotensin-converting enzyme (ACE). The nonclassical pathway, mediated instead by ACE-2, involves the conversion of Ang II into angiotensin 1–7 (Ang 1–7) and Ang I into angiotensin 1–9 (Ang 1–9). Ang II on AT1 receptors has a vasoconstrictive, inflammatory, and profibrotic function. The RAS-mediated effects of Ang (1–7) and Ang (1–9) are anti-inflammatory, vasodilatory, and antifibrotic [24]. In various diseases, including bacterial infections, the dysregulation and dysfunction of the RAS can lead to worsening pathology and organ injury [25,26,27]. In particular, the RAS is involved in pathological infections because it regulates the immune response, and dysregulation of the system’s function can be responsible for and lead to organ injury due to the hyperinflammatory state, which can occur in bacterial sepsis that occurs in pneumonia [28]. From the literature data, it is certain how RAS can be responsible for worsening pathology and organ damage [28]. In fact, it is known that RAS activation has a different function in certain conditions, such as bacterial and pulmonary diseases and asthma, as well as in smokers, demonstrating how the system has a close link to the proper physiology of the lungs and airways. Evidence in the literature suggests that, at the level of ACE/ACE-2, the balance and physiological homeostasis of the RAS are likely to be altered by infection, resulting in pathology. This RAS dysfunction probably plays an important role in organ damage and the activation of a hyperinflammatory process during the septic and infectious state. These aspects are yet to be fully elucidated, however. In this direction, the regulation of RAS and the ACE/ACE-2 balance would lead to a way to moderate organ damage during sepsis from infectious disease [29]. Furthermore, it has been demonstrated and is well understood that ACE-2 plays a crucial role in fibrogenesis and inflammation in various tissues and organs, including the lungs. It is very likely that ACE-2 has a protective effect, and its decrease may cause the inflammatory state of the airways and lungs to worsen. In addition, specific studies in the literature show that ACE-2 is decreased in infectious diseases, pulmonary fibrosis, and chronic obstructive pulmonary disease. It should also be noted that Ang II, Ang 1–7, and Ang 1–9 have different physiological and biological effects. In fact, the physiological effects of Ang II are vasoconstriction and stimulating aldosterone production and release; but they can also cause cardiac damage, myocardial hypertrophy, endothelial dysfunction, interstitial fibrosis, increased inflammatory status, and increased coagulation. If altered, these effects can lead to serious complications in a patient with an infection [30]. Ang II can cause increased inflammation through the production of IL-6, TNF (Tumor Necrosis Factor), and other inflammatory cytokines [19]. However, it is important to note that all of these biological effects are mediated by angiotensin AT-1 (AT-1r). ACE-2 can lead to a reduction in the adverse effects of Ang II through several mechanisms, such as the conversion of Ang II to Ang 1–7. Ang1–7 has opposite effects to Ang II through the Mas receptor (MASr) and Ang II type II receptors (AT-2r). The numerous effects of the RAS suggest that its regulation could be very useful in infectious diseases, such as bacterial pneumonia [31]. Finally, disorders of the RAS can be caused by viral [32,33,34] or neoplastic diseases, such as those affecting the kidneys [35] or multiple organs [36,37].

## 5. Vitamin D and RAS

Clinical and epidemiological studies have repeatedly highlighted the importance of vitamin D in the RAS at the clinical, molecular, and pathophysiological levels. The relationship between vitamin D, blood pressure, and plasma renin has been discussed in the literature in many studies [38,39]. Data show that very low plasma levels of 25-hydroxyvitamin D can decrease the up-regulation of RAS in physiologically healthy humans [40,41,42]. Mechanistic studies have been published and confirm that the negative regulation of renin by calcitriol:renin expression and plasma angiotensin II production have been greatly increased in mice lacking the calcitriol receptor (VDR-null), resulting in cardiac hypertrophy, hypertension, and increased water intake. The inhibition of 1,25-dihydroxyvitamin-D(3) synthesis in wild-type mice also led to increased renin expression, whereas the injection of 1,25-dihydroxyvitamin-D(3) led to renin regression and suppression [43]. The optimal vitamin D concentration status for use in different therapeutic and clinical settings, including patients affected by bacterial infections, has not yet been well studied; how the RAS and cholecalciferol interact, as well as at which concentration of vitamin D, is currently under study. Long-term vitamin D deficiency is presumed to activate the RAS, decreasing lung functioning, resulting in increased risk of diseases, such as bacterial infections or fibrosis [44]. Bacterial infections are more severe in vulnerable individuals, such as elderly, diabetic, hypertensive, obese, and smoking patients. The RAS functioning system in these categories of patients is impaired. Therefore, the normalization of cholecalciferol concentrations and regularization of RAS functioning with the transcriptional suppression of renin gene expression could be helpful in some clinical conditions, such as in acute lung injury (ALI) and acute respiratory distress syndrome (ARDS) due to bacterial infections [45].

**Figure 1:**
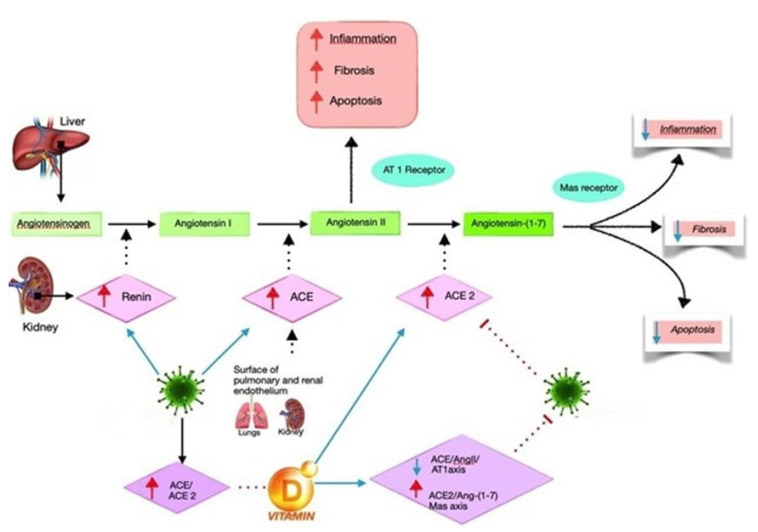
Plausible vitamin D mechanism of action as a negative endocrine modulator of the renin–angiotensin system (RAS). It can induce ACE2/Ang-(1–7)/MasR axis activity and inhibit renin and the ACE/Ang II/AT1R axis, thereby increasing the expression and concentration of ACE2, MasR, and Ang-(1–7) and playing a potential protective role against infectious disease with a reduction of fibrosis and inflammatory effects.

Evidence from the literature has shown how cholecalciferol can exert a suppressive action on ACE, renin, and Ang II expression by increasing the ACE2 levels in LPS-induced ALI [46]. Consequently, vitamin D can diminish LPS-stimulated ALI through the induction of the ACE2/Ang-(1–7) axis and the suppression of the axis of renin and ACE/Ang II/AT1R [47]. A second study on animal models showed that, in the first group, 1,25(OH)2D3 increased the expression of ACE2 and VDR mRNA of LPS-induced ALI compared with controls, assuming that the major expression of ACE2 and VDR mRNA plays a crucial role in the protection of ALI proliferation [48]. Figure 1 illustrates the efficacy of cholecalciferol hypothesized for the treatment of ARDS-inducing bacterial infections. There is some evidence in the literature in reference to potentially beneficial effects of RASS-mediated regulation; notwithstanding, it is more likely that any protective effect of cholecalciferol against pathogenic bacteria is related to the reduction of both the cytokine response and ARDS severity/risk ratio [49].

## 6. Conclusions and Future Perspectives

Compared with a study that has recently been published in the literature [17], it is noteworthy that the use of vitamin D may be an interesting therapeutic proposal as an adjunct to standard therapy for the treatment of acute childhood pneumonia. At the same time, there is evidence concerning the action of vitamin D in the restoration of RAS functioning. Based on this evidence, our review may suggest a possible use for vitamin D as a supportive therapeutic strategy to treat specific infections of bacterial origin. In fact, by triggering the cytokine cascade and the inflammatory response, the bacterial infection could damage the response of the RAS; consequently, it is conceivable that the regulation of RAS by vitamin D as a supportive treatment could be a potential therapeutic approach to counteract the expansion of ARDS induced by bacterial pathology and to reduce the hyperinflammatory and hyperfibrotic state responsible for organ damage under bacterial sepsis conditions. Further preclinical and clinical studies are ongoing and are needed to further investigate this interesting field of research to develop new evidence and to provide additional information.

## Data Availability

Not applicable.
